# Vertical Distribution and Controlling Factors Exploration of Sc, V, Co, Ni, Mo and Ba in Six Soil Profiles of The Mun River Basin, Northeast Thailand

**DOI:** 10.3390/ijerph17051745

**Published:** 2020-03-07

**Authors:** Wenxiang Zhou, Guilin Han, Man Liu, Chao Song, Xiaoqiang Li, Fairda Malem

**Affiliations:** 1 Institute of Earth Sciences, China University of Geosciences, Beijing 10083, China; zhouwenxiang@cugb.edu.cn (W.Z.); lman@cugb.edu.cn (M.L.); xiaoqli@cugb.edu.cn (X.L.); 2 Institute of Hydrogeology and Environmental Geology, Chinese Academy of Geological Sciences, Shijiazhuang 050061, China; songchao@mail.cgs.gov.cn; 3 Environmental Research and Training Center, Department of Environmental Quality Promotion, Klong 5, Klong Luang, Pathumthani 12120, China; mfairda@yahoo.com

**Keywords:** soil heavy metals, enrichment factor, geoaccumulation index, Mun River Basin, Northeast Thailand

## Abstract

Exploring the enrichment and controlling factors of heavy metals in soils is essential because heavy metals can cause severe soil contamination and threaten human health when they are excessively enriched in soils. Soil samples (total 103) from six soil profiles (T1 to T6) in the Mun River Basin, Northeast Thailand, were collected for the analyses of the content of heavy metals, including Sc, V, Co, Ni, Mo, Ba. The average contents of soil heavy metals decrease in the following order: Ba, V, Ni, Sc, Co, and Mo (T1, T3, T4 and T5); Ni, V, Ba, Co, Sc, Mo, and Ba (T2); Ba, V, Sc, Ni, Mo, and Co (T6). An enrichment factor (EF) and geoaccumulation index were calculated to assess the degree of heavy metal contamination in the soils. The EFs of these heavy metals in most samples range from 0 to 1.5, which reveals that most heavy metals are slightly enriched. Geoaccumulation indexes show that only the topsoil of T1 and T2 is slightly contaminated by Ba, Sc, Ni, and V. Soil organic carbon (SOC), soil pH and soil texture are significantly positively correlated with most heavy metals, except for a negative correlation between soil pH and Mo content. In conclusion, the influence of heavy metals on soils in the study area is slight and SOC, soil pH, soil texture dominate the behavior of heavy metals.

## 1. Introduction

Soil is a huge sink for heavy metals derived from parent rocks and anthropogenic activities, most of which will cause serious contamination once enriched in soils [1]. Because air, water and rocks interface in soil environment, the heavy metals in soils are transported by various carriers, which may lead to the enrichment of heavy metals in some areas [2,3,4]. The Mun River Basin is located in Northeast Thailand, which is one of the largest agricultural regions in Thailand [5]. The enrichment of toxic heavy metals will lower soil fertility and increase the input of these heavy metals to the food chain, which may be adverse to human health [2]. Therefore, it is highly significant to study the distribution characteristics of heavy metals in the Mun River Basin. 

The heavy metals Co, Ni, Ba and V are considered as important indicators of soil toxicology, variability in soil composition and human health risks [6]. With the urbanization and industrialization in the Mun River Basin, these heavy metals are frequently emitted out from tire abrasion, industrial production, and fertilizer application and can easily accumulate in soils [6]. The Mun River Basin is an important agricultural region in Thailand and large amounts of fertilizers are used, so soils in the basin are easily polluted, making it essential to explore the distributions of these heavy metals in soil profiles. Scandium (Sc) has drawn great attention due to its potential contamination to the environment and the risks to cause various diseases with its wide application in medicine and technology [7], so it is significant to explore the potential pollution of Sc emitted out from factories and hospitals across the basin. In the last three decades, considerable urbanization and industrialization have occurred in Thailand, which may cause soil contamination of these heavy metals by the combustion of fossil fuel, emission of vehicle exhaust, etc. [6,8]. Although the concentrations of molybdenum (Mo) are usually quite low in soils and rocks, Mo is essential for plants, animals and humans [9]. Relative to soil contamination problems, Mo deficiency has attracted more attention because it will largely reduce crop yield [10]. Mo deficiency in the crops grown in Northeast Thailand has been reported, making it necessary to study the distribution of Mo there [11,12]. Moreover, as the most important agricultural region in Thailand, heavy metal contamination or deficiency would affect human health through the food chain. Therefore, these six heavy metals (Sc, V, Co, Ni, Mo, Ba) were selected to help to assess the soil condition and provide the basis for the strategies of agricultural management.

The mobility of soil heavy metals is controlled by various factors, such as soil pH, soil organic matter (SOM) and soil texture [2,13]. Generally, the more mobile heavy metals are, the more available they are for plants, which increases the risk that they enter the food chain of humans. The relationships between these heavy metals and soil properties may vary with different soil conditions, for example, the adsorption of Mo on clay is regulated by soil pH [9]. It is therefore necessary to explore the relationships between heavy metals and soil properties to better understand the distributions of these heavy metals. This study aims to: (1) learn about the spatial distributions of these heavy metals in six soil profiles; (2) assess the risks of soil heavy metal contamination; and (3) explore the controlling factors of soil heavy metal behaviors. 

## 2. Materials and Methods 

### 2.1. Study Area

**Figure 1:**
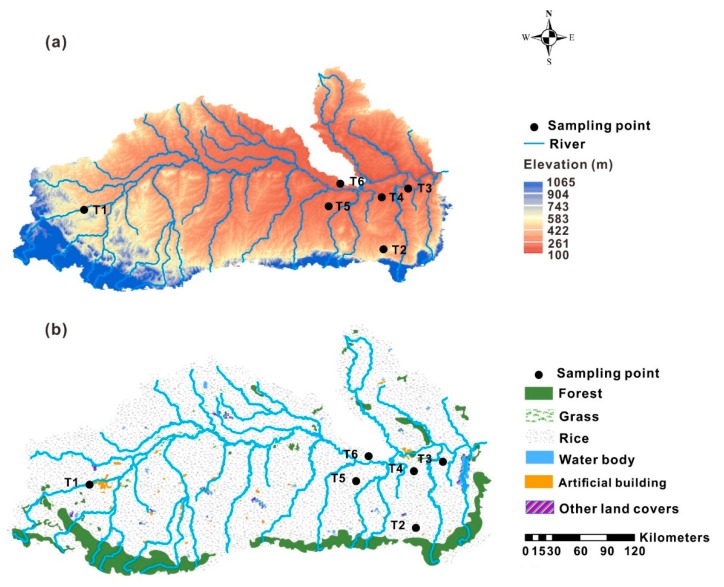
Map of elevation (**a**) and land cover types (**b**) in the Mun River Basin (Data of elevation come from the website: https://opentopography.org/start).

This study was conducted in the Mun River Basin, the largest river basin (82,000 km^2^) in Northeast Thailand (14°00′~16°00′ N, 101°30′~105°30′ E). The Mun River Basin, of which the elevation ranges from 100 to 1065 m (Figure 1), is dominated by a tropical savanna climate which leads to a dry season and a rainy season all year round [14]. Most crops are cultivated during the rainy season (April to November) when 85%–90% of the annual precipitation falls [15]. Alluvial and sandy soils in the basin are heavily leached and have low organic matter contents, leading to the frequent use of fertilizers [15,16]. Land cover types are various in the basin, including crops, forest, grass, water, etc., and most forest lands are distributed near the southwestern and southeastern border of the basin (Figure 1). In the Mun River Basin, shale, siltstone, sandstone, and conglomeritic sandstone are the main rock types of the rock basement which is covered by the Quaternary alluvial sediment [17]. 

### 2.2. Sampling and Analysis

Soil samples (total 103) were collected in March 2018, from six soil profiles in the Mun River Basin. These soil profiles were dug on paddy lands (T1, T3), forest lands (T2, T6), wetlands (T4) and built-up lands (T5), respectively. Soil samples were collected at intervals of 10 cm and the number of soil samples was 22, 5, 10, 20, 41, and 5 in the six soil profiles, respectively. These soil profiles are chosen because they are under the main land-use types in the Mun River Basin and are distributed across the basin (Figure 1). The distribution of heavy metals in these profiles would reflect the general condition of heavy metals in the Mun River Basin. These soil profiles are described in detail in Table 1.

Soil samples were air-dried at 25 ℃ and unwanted materials such as stones and plant roots were removed by passing soil samples through a 2 mm sieve [18,19]. Soil samples were ball milled thoroughly to pass a 200-mesh sieve (<75 μm) with the Retsch MM400 (Retsch GmbH, Haan, Germany), a widely used grinding machine [20]. 100 mg soil powders were digested in Teflon beakers using ultra-pure HNO_3_ (70%) and ultra-pure HF (40%) (3:1) at 120 ℃ for seven days [21]. If some organic solid residue remains undissolved, aqua regia (HNO_3_: HCl, 1:3) would be used to remove organic matters. When all samples were dissolved, the digestion process was repeated twice using 50 ml ultra-pure HNO_3_ (70%) to break down potential fluorine compounds [21]. After these treatments, the prepared samples were diluted in ultra-pure HNO_3_ (2%) of about 50 ml. Finally, the concentration of the heavy metals Co, Ni and Mo were measured by ICP-MS (Elan DRC-e, Perkin Elmer, Waltham, MA, USA) and the concentrations of the heavy metals Ba, Sc, V and Ti were measured by ICP-OES (Optima 5300DV, Perkin Elmer, Waltham, MA, USA).

### 2.3. Data Treatment

The enrichment factor (EF) index and the geoaccumulation index (*I_geo_*) are two important methods to assess the enrichment and contamination of heavy metals in soils [22,23]. The upper continental crust (UCC) is usually chosen as the reference material for the calculation of both indexes [1]. All background values were provided by Wedepohl [24]. 

For the enrichment factor, Ti was used as a reference element due to its characteristic of low occurrence variability [25]. The enrichment factor was calculated according to the following formula [26,27]:

EF = ([M]/[Ti])_S_/([M]/[Ti])_UCC_,

where M represents heavy metal concentrations and S refers to soil samples.

Based on the EF values, the enrichment of heavy metals can be categorized into five grades: minimal enrichment (EF ≤ 2); moderate enrichment (2 < EF < 5); significant enrichment (5 ≤ EF < 20); high enrichment (20 ≤ EF < 40); and extremely high enrichment (EF ≥ 40) [28].

The geoaccumulation index was calculated as follows [29]:

*I_geo_* = log_2_(C_n_/1.5B_n_),

where C_n_ represents the concentration of the heavy metal n in soil samples; B_n_ represents the concentration of the heavy metal n in reference material (UCC); and the factor 1.5 is used to eliminate the lithogenic effects [1]. According to the *I_geo_* values, soil contamination can be categorized into seven classes from uncontaminated (*I_geo_*< 0) to very highly contaminated (*I_geo_*> 5) [22].

### 2.4. Statistical Analysis

In this study, Pearson correlation analysis was applied to analyze the relationship between SOC, soil pH, clay and soil heavy metals. Principal component analysis (PCA) was applied to identify the sources of soil heavy metals. All mathematical analyses were done by SPSS 20 (Statistical Package for Social Science, IBM, Armonk, NY, USA). Figure 1 was produced by ArcMap 10.0 software and other figures were produced by OriginPro 9.0 software (OriginLab Corporation, Northampton, MA, USA).

## 3. Results and Discussion

### 3.1. Contents of Heavy Metals in Soil

**Figure 2:**
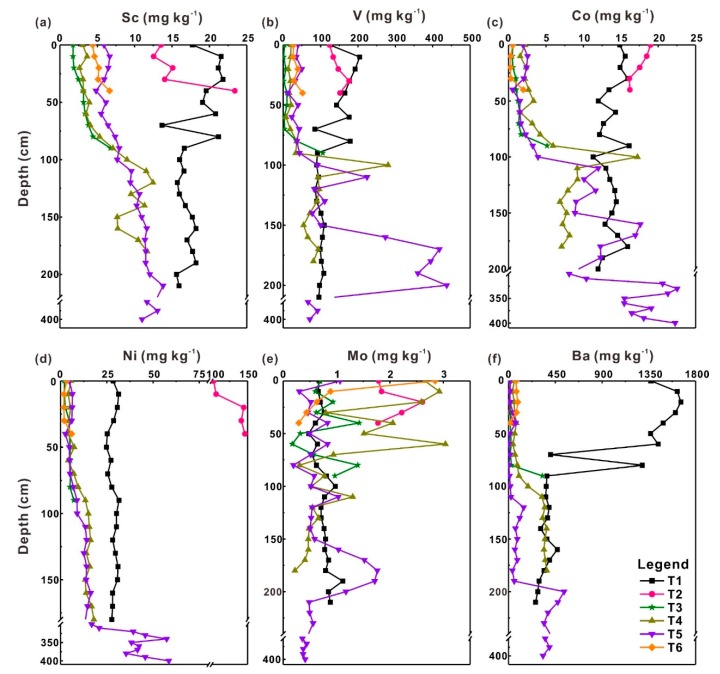
Vertical distributions of heavy metals, including Sc (**a**), V (**b**), Co (**c**), Ni (**d**), Mo (**e**) and Ba (**f**). (Some data were omitted because of their small variation ranges).

The profile distributions of six heavy metals (Sc, V, Co, Ni, Mo, Ba) in six profiles under four land-use types are shown in Figure 2. The average contents of heavy metals in samples from soil profiles T1, T3, T4 and T5 decrease in the following sequence: Ba > V > Ni > Sc > Co > Mo (Table 2). In soil profiles T2 and T6, these contents are in the following sequences respectively: Ni > V > Ba > Co > Sc > Mo and Ba > V > Sc > Ni > Mo > Co (Table 2). 

Vertically, the contents of heavy metals generally increase with depth in most soil profiles, excluding profile T1, which is quite marked in Sc, V, and Co (Figure 2). Most rainfall in the study area is mainly concentrated in the rainy season [15], which causes a large amount of precipitation in a short period and results in severe leaching of heavy metals down these soil profiles [30]. The vertical distributions of heavy metal concentrations can reflect the relative mobility of these metals in soil profiles [3]. Based on the distribution characteristics of these heavy metals in the six profiles (Figure 2), it can be concluded that the mobility of heavy metals Sc, V, and Co is higher than the mobility of Ni, Mo, and Ba in these soil profiles. Soil profile T1 is dug in rice fields where fertilizer is frequently used. The higher concentrations of these heavy metals in the topsoil of profile T1 are caused by the applications of pesticide and fertilizer [23]. Huge positive peaks of heavy metal concentrations (V, Co, Mo) in soil profile T5 were observed at approximately 165–200 cm depth. According to the field recordings, there is a layer of iron-manganese oxides at 160–205 cm depth, where these positive peaks occur (Table 1). The phenomenon is attributed to the adsorption of iron–manganese oxides on these heavy metals [31,32]. Due to the surface complex formation and the existence of specific adsorption potentials, iron–manganese oxides own a relatively strong ability to absorb and concentrate heavy metals in soils [31,33]. However, heavy metals Sc, Ni and Ba do not show positive peaks of concentrations in that layer, indicating that the influence of iron–manganese oxides on these heavy metals is limited. Potential reasons behind the phenomenon need to be further explored. 

### 3.2. Assessment of Soil Heavy Metal Contamination

#### 3.2.1. Enrichment of heavy metals

**Figure 3:**
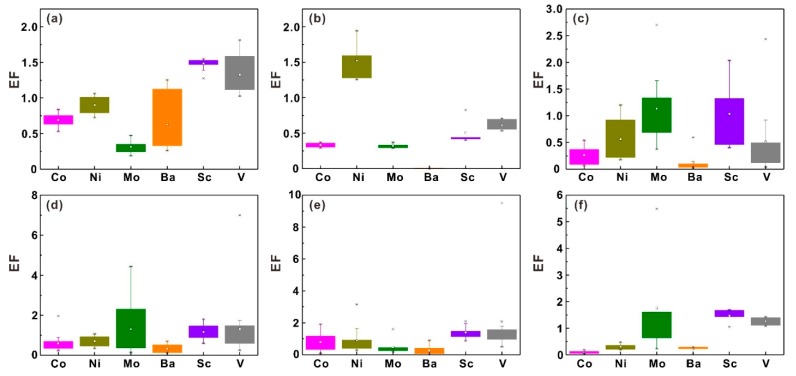
Box plots of heavy metal EF values in six soil profiles, including soil profiles T1 (**a**), T2 (**b**), T3 (**c**), T4 (**d**), T5 (**e**) and T6 (**f**).

The calculated EF factor values of most samples are lower than 2 (Figure 3), which indicates the minimal enrichment of these heavy metals in soils [28]. Several extremely abnormal EF values occur in soil profile T5, exactly at the depth where abnormal heavy metal concentrations appear (Figure 2), which can be attributed to the absorption by iron–manganese oxides. The sources of heavy metals in soil profiles comprise crustal materials (e.g. parent rocks) and non-crustal materials (e.g. anthropogenic sources). Due to lithogenic effects, an EF value = 1.5 is regarded as an important threshold for assessing whether human activities influence heavy metal concentrations in soils [34]. Except for the V in the soil profile T5 and Mo in the soil profile T6, the average EF values of these heavy metals in different soil profiles are lower than 1.5 which indicates that these heavy metals are mainly derived from natural processes [23,35]. The EF values of V (2.2) and Mo (1.8) in soil profiles T5 and T6 are higher than 1.5, indicating that non-crustal materials are also important contributors for these heavy metals [35]. The EF values of Mo in soil profile T1 that was dug in paddy fields are lower than 0.5 which shows that the Mo deficiency may have occurred in local agricultural lands. Molybdenum fertilizer may be a good choice to make up the Mo deficiency in local agricultural lands [11].

#### 3.2.2. Contamination Assessment by I_geo_ Values in Topsoil.

**Figure 4:**
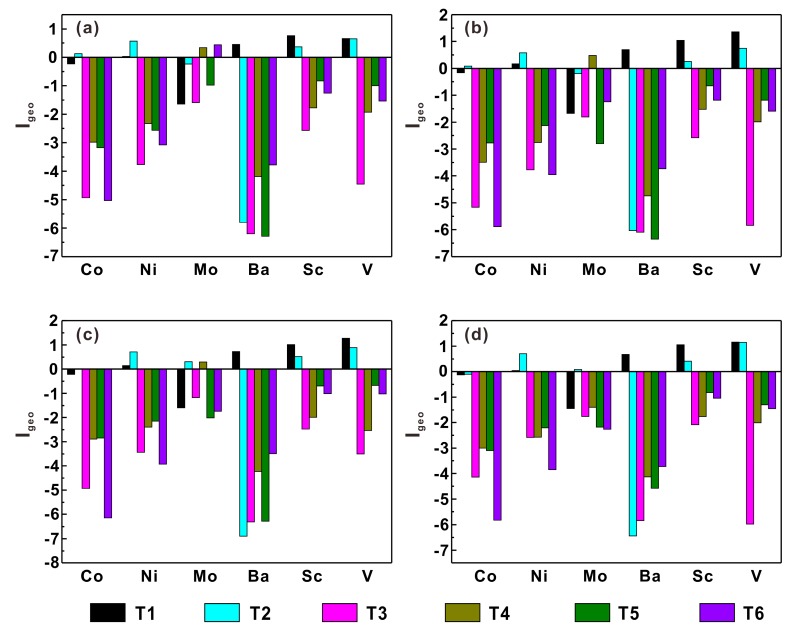
Geoaccumulation indexes of heavy metals in the surface layers of six soil profiles. 0–10 cm (**a**); 10–20 cm (**b**); 20–30 cm (**c**); 30–40 cm (**d**).

Heavy metal contamination was reported to be most obvious in topsoil (0–40 cm) [36], so the contamination assessment should be concentrated on topsoil. The *I*_geo_ values of these heavy metals in topsoils (0–10, 10–20, 20–30, 30–40 cm) of six soil profiles are presented in Figure 4. These values of most metals are lower than 0 at all depths of all soil profiles, except for Sc and V in soil profiles T1 and T2 (Figure 4). The results show that the soils of the six soil profiles are not polluted by these heavy metals in the course of human activities. The positive *I*_geo_ values of heavy metals mainly occur in the topsoil of soil profiles T1 and T2, which are located in a paddy field and oak forest, respectively. The *I*_geo_ values of Ba and Sc in profile T1 and Ni, Sc, and V in profile T2 are roughly in the range of 0–1, indicating that the topsoil of these two soil profiles is slightly polluted by these heavy metals. Except for the surface soil layer (0–10 cm), the *I*_geo_ values of V are in the range of 1–2, indicating the moderate contamination of V in the topsoil of soil profile T1. Heavy metals that may be adverse for human health, such as Ni and V, have been reported to accumulate in soils accompanied by the frequent application of fertilizers [37], so a potential reason for the contamination is the application of fertilizers. Chemical fertilizers cause heavy metal pollution by directly inputting various heavy metals into soils, while organic fertilizers enhance heavy metal pollution levels by forming complexes with heavy metal ions.

### 3.3. Correlation Analysis Between Soil Heavy Metals and Soil Properties.

#### 3.3.1. Characteristics of Inter-element Relationships

The results of Pearson correlation analysis between soil heavy metals and the three soil properties (SOC, soil pH and clay contents) are presented in Table 3. The inter-element relationships show that all heavy metals are closely correlated (*P* < 0.01, *P* < 0.05) (except for Mo–Co, Mo–Sc, Mo–V and Ba–Ni), which indicates that the heavy metals Co, Ni, Ba, Sc and V are derived from similar natural processes and have similar geochemical behavior [23,38]. The reason Mo is distinct from other heavy metals may be that Mo usually forms oxyanions but not cations in soil and rarely coordinates with ligands such as OH^-^ and Cl^-^ like most heavy metals [39]. 

#### 3.3.2. Influence of Soil Organic Matter on Heavy Metals

**Figure 5:**
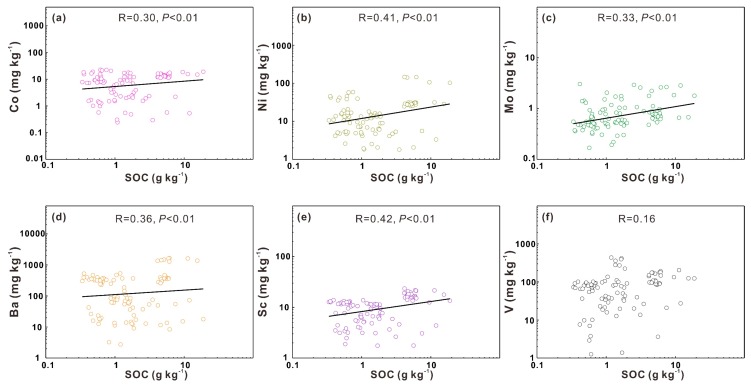
Linear correlation between soil organic carbon (SOC) and the heavy metals Co (**a**), Ni (**b**), Mo (**c**), Ba (**d**), Sc (**e**), V (**f**).

The mobility of heavy metals is reported to be controlled by the process of adsorption and desorption in soils, which is affected by some soil properties such as soil organic matter, soil pH and clay contents [13,40,41]. Soil organic matter can be converted into SOC contents with a conversion factor, making SOC contents an important measurement for SOM [42]. Except for V, these heavy metals are significantly positively correlated with SOC contents (*P* < 0.01), indicating that organic matter benefits the accumulation of heavy metals (Figure 5). The heavy metal V occurs in various oxidation states (II, III, IV, or V), and mainly exists as V^5+^ under weathering conditions [9]. Because SOM is equipped with numerous negatively charged groups, it owns a strong ability to absorb heavy metal divalent and trivalent cations [21]. From this perspective, the weak relationship between V and SOC contents can be explained by the higher valence. However, while the main oxidation states of Mo in the lithosphere are Mo(IV) and Mo(VI), Mo is still closely correlated with SOC contents (*P* < 0.01). The phenomenon may demonstrate that Mo is stabilized by organic matter in a different mechanism, such as complexing with catechol groups of tannin-like compounds in soil organic matter [43]. 

#### 3.3.3. Influence of Soil pH on Heavy Metals

**Figure 6:**
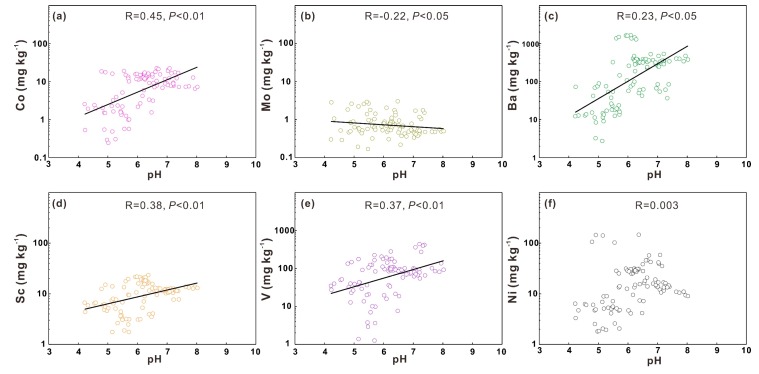
Linear correlation between soil pH and the heavy metals Co (**a**), Mo (**b**), Ba (**c**), Sc (**d**), V (**e**), Ni (**f**).

Soil pH is widely regarded as the most important factor influencing the mobility and solubility of soil heavy metals [13,44]. Except for Ni, all heavy metals are significantly influenced by soil pH (*P* < 0.01 for Co, Sc, and V; *P* < 0.05 for Mo and Ba) (Figure 6). Soil heavy metals Co, Sc, Ba and V are positively correlated with soil pH while Mo is negatively correlated with soil pH. The positive relationship between heavy metals and soil pH has been widely reported [9,13,23]. Soil pH regulates heavy metal contents by changing the available heavy metal contents [45,46]: higher pH means more OH^-^ existing in soil environment, which would increase the fixation of most heavy metals and reduce the amount of heavy metals uptake by plants, thus benefitting the accumulation of heavy metals. The existing form of Mo in soils results in the negative correlation between Mo and soil pH: Mo always forms oxyanions instead of cations like most heavy metals [9]. In this case, Mo is more available and mobile under higher pH conditions which corresponds to the negative relationship between Mo and soil pH. The soil pH values of these samples are in the range of 5.6–7.2 for most samples (around 75%) [47]. The heavy metal Ni is reported to be most sensitive to soil pH when the soil pH ranges from 5.0–5.5 [48], so the weak relationship between Ni and soil pH may result from the ranges of soil pH values. 

#### 3.3.4. Influence of Soil Texture on Heavy Metals

**Figure 7:**
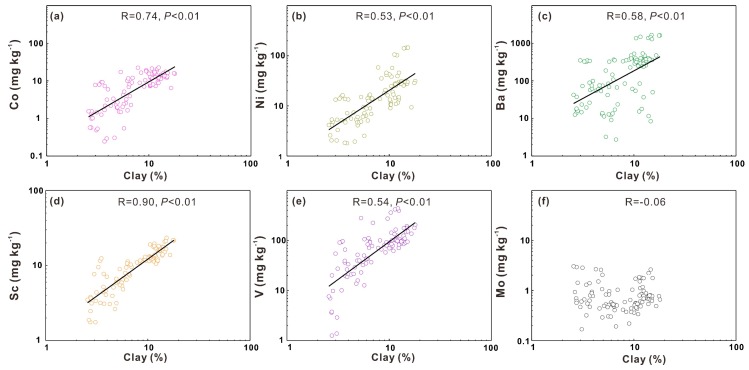
Linear correlation between clay contents and the heavy metals Co (**a**), Ni (**b**), Ba (**c**), Sc (**d**), V(**e**), Mo (**f**).

Soil texture is a term used to describe the distributions of soil particle sizes in soils [49]. According to the diameter of soil particles, they can be categorized into three groups: clay, silt and sand. All heavy metals (excluding Mo) are significantly correlated (*P* < 0.01) with clay contents in soils (Figure 7), indicating the important role of clay in stabilizing soil heavy metals. Finer soil particles are reported to have a stronger ability to absorb and fix heavy metals on their surface because of their bigger surface area and various functional groups [1,50,51,52]. Moreover, clay is also found to stabilize soil organic matter that would also fix heavy metals [13,47]. 

Therefore, the clay benefits the accumulation of soil heavy metals. However, relatively high soil pH would prevent the adsorption of molybdate by clay [53], which provides a potential explanation for the weak relationship between Mo and clay contents.

### 3.4. Principal Component Analysis for Heavy Metals

Principal component analysis (PCA) is an important multivariate analysis method applied to identify the sources of soil heavy metals [54]. The prerequisite of the application of PCA is to pass the Kaiser–Meyer–Olkin (KMO) and Bartlett’s sphericity tests: the KMO measure should be higher than 0.5 and Bartlett’s sphericity test should be significant (*P* < 0.001) [28,55,56]. The KMO value equals 0.61 and Bartlett’s sphericity test is significant (*P* = 0) in this study, indicating that PCA is applicable to the analysis of these soil samples. The results of PCA are presented in Table 4. 

On condition that eigenvalues are higher than 1, two principal components are extracted which explain 72.41% of the total variance (PC1: 49.70%; PC2: 22.71%) (Table 4). As shown in Table 4, PC1 is positively correlated with the heavy metals Co, Ni, Ba, Sc and V, and PC2 positively correlates with Mo. Based on the results of PCA, these heavy metals can be categorized into two groups (PC1: Co, Ni, Ba, Sc and V; PC2: Mo). The heavy metals Co, Ni, Sc and V are usually regarded as lithophile elements that are associated with soil minerals [3,52], while Mo and Ba are regarded as anthropogenic elements [52]. As an important agricultural region, anthropogenic sources in the Mun River Basin should be the application of fertilizers, including chemical fertilizers and organic fertilizers as mentioned before. Therefore, the first factor may refer to soil mineral compositions which are largely determined by the parent rocks and the second factor may refer to the impact of human activities. In this study, only the heavy metal Mo is dominated by the second factor, indicating that soil in the study area is little influenced by the heavy metals Co, Ni, Ba, Sc and V derived from anthropogenic activities. Although the topsoil seems to be slightly or moderately polluted by these heavy metals due to the application of fertilizers as discussed before, the impact of anthropogenic activities is relatively limited compared with that of natural processes according to the PCA results. 

## 4. Conclusions

The soil heavy metal concentrations decrease following the sequence of Ba, V, Ni, Sc, Co and Mo in soil profiles T1, T3, T4 and T5; the sequence of Ni, V, Ba, Co, Sc, Mo in soil profile T2; and the sequence of Ba, V, Sc, Ni, Mo, Co in soil profile T6. The EF values of most samples are lower than 1.5, indicating that these heavy metals are mainly derived from natural processes. The EF values of Mo in paddy fields are lower than 0.5, which provides evidence for the existence of Mo deficiency in the agricultural lands of Northeast Thailand. The application of molybdenum fertilizer should be advocated to eliminate the adverse effect of Mo deficiency on crops. The geoaccumulation index shows that only the topsoil of the soil profiles T1 and T2 is slightly contaminated by the heavy metals Ba, Sc, Ni and V. Overall, soils in the study area are in relatively good condition. The PCA results reveals that two factors (parent rocks and human activities) are responsible for 72.4% of the total variations of heavy metals in soil profiles: Co, Ni, Ba, Sc and V are dominated by the first factor while Mo is dominated by the second factor. The correlation analysis shows that soil organic matter and clay benefit the accumulation of heavy metals. Soil pH is positively correlated with heavy metals Co, Ba, Sc and V but is negatively correlated with Mo, which may result due to Mo usually existing in the form of oxyanions instead of cations like most heavy metals. 

## Figures and Tables

**Table 1:** General description of the sampling information.

Sampling Point	Land Cover	Depth (cm)	Field Description
T1	Crop	220	Relatively uniform at all depths
T2	Oak forest	50	Compact soil; brown red
T3	Crop	100	0–12 cm: Black root layer
			12–100 cm: Fine silt
			>100 cm: Iron manganese nodules
T4	Grass	193	0–20 cm: Root layer
			20–112 cm: Fine silt
			112–180 cm: Weathering crust
			180–193 cm: Gray and green sand
			>193 cm: Bedrock
T5	Artificial building	405	0–105 cm: Fine silt
			105–205 cm: Iron manganese nodules
			205–405 cm: Clay layer
T6	Forest	50	Fine silt

**Table 2:** Statistical results for heavy metal contents (mg kg^−1^) of soil samples from six soil profiles.

Soil Profile	Item	Sc	V	Co	Ni	Mo	Ba
T1	Min	13.65	84.65	11.30	24.76	0.48	259.62
	Max	21.82	204.50	16.12	31.58	1.12	1659.68
	Mean (SD)	17.95 (2.27)	123.47 (39.68)	13.70 (1.50)	28.58 (1.97)	0.75 (0.14)	764.44 (561.23)
T2	Min	12.54	124.26	16.15	102.61	1.77	8.41
	Max	23.40	176.18	18.98	146.22	2.61	49.94
	Mean (SD)	15.72 (4.39)	146.64 (19.79)	17.47 (1.28)	128.15 (21.88)	2.05 (0.36)	20.62 (16.79)
T3	Min	1.76	1.26	0.49	2.05	0.17	12.57
	Max	6.76	105.87	5.15	7.14	1.42	324.63
	Mean (SD)	3.26 (1.53)	18.22 (32.50)	1.45 (1.36)	4.41 (1.66)	0.77 (0.41)	48.62 (97.07)
T4	Min	2.63	7.64	1.55	4.10	0.22	37.43
	Max	12.46	280.59	17.23	18.00	3.04	367.81
	Mean (SD)	6.89 (3.52)	59.75 (62.29)	5.78 (3.89)	10.72 (5.10)	1.19 (0.97)	188.94 (142.42)
T5	Min	4.83	12.72	0.55	2.53	0.19	2.75
	Max	13.88	437.43	22.55	58.55	1.76	534.58
	Mean (SD)	10.45 (2.67)	111.98 (107.71)	10.05 (6.45)	18.35 (15.25)	0.65 (0.36)	202.71 (171.92)
T6	Min	4.38	26.37	0.24	1.80	0.29	13.86
	Max	6.66	51.76	1.97	6.01	2.85	89.26
	Mean (SD)	5.19 (0.89)	34.75 (10.79)	0.67 (0.74)	2.98 (1.81)	1.02 (1.05)	65.37 (29.51)

**Table 3:** Correlation analysis between heavy metals and soil properties.

	Co	Ni	Mo	Ba	Sc	V	SOC	pH	Clay
Co	1	0.652 **	−0.063	0.419 **	0.763 **	0.558 **	0.298 **	0.451 **	0.739 **
Ni	0.652 **	1	0.270 **	0.083	0.510 **	0.248 *	0.407 **	0.003	0.530 **
Mo	−0.063	0.270 **	1	−0.239 *	−0.155	0.115	0.329 **	−0.223 *	−0.064
Ba	0.419 **	0.083	−0.239 *	1	0.701 **	0.321 **	0.360 **	0.225 *	0.575 **
Sc	0.763 **	0.510 **	−0.155	0.701 **	1	0.517 **	0.414 **	0.378 **	0.897 **
V	0.558 **	0.248 *	0.115	0.321 **	0.517 **	1	0.159	0.365 **	0.543 **
SOC	0.298 **	0.407 **	0.329 **	0.360 **	0.414 **	0.159	1	−0.298 **	0.433 **
pH	0.451 **	0.003	−0.223 *	0.225 *	0.378 **	0.365 **	−0.298 **	1	0.357 **
Clay	0.739 **	0.530 **	−0.064	0.575 **	0.897 **	0.543 **	0.433 **	0.357 **	1

* Correlation is significant at the 0.05 level. ** Correlation is significant at the 0.01 level

**Table 4:** Principal component analysis (PCA) of heavy metals.

Element	Component	
	PC1	PC2
Co	0.90	0.11
Ni	0.64	0.56
Mo	−0.05	0.85
Ba	0.66	−0.52
Sc	0.93	−0.17
V	0.68	0.13
Eigenvalue	2.98	1.36
Variance %	49.70	22.71
Cumulative %	49.70	72.41
